# Relaxation – Induced by Vibroacoustic Stimulation via a Body Monochord and via Relaxation Music – Is Associated with a Decrease in Tonic Electrodermal Activity and an Increase of the Salivary Cortisol Level in Patients with Psychosomatic Disorders

**DOI:** 10.1371/journal.pone.0170411

**Published:** 2017-01-23

**Authors:** Hubertus Sandler, Uta Fendel, Petra Buße, Matthias Rose, Rainer Bösel, Burghard F. Klapp

**Affiliations:** 1 Department for General Internal and Psychosomatic Medicine, Charité Universiätsmedizin Berlin, Berlin, Germany; 2 International Psychoanalytic University Berlin, Berlin, Germany; 3 Department of Cognitive Neuroscience, Freie Universität Berlin, Berlin, Germany; University of Marburg, GERMANY

## Abstract

Vibroacoustic stimulation by a Body Monochord can induce relaxation states of various emotional valence. The skin conductance level (SCL) of the tonic electrodermal activity is an indicator of sympathetic arousal of the autonomic nervous system and thus an indicator of the relaxation response. Salivary cortisol is considered to be a stress indicator of the HPA-axis.

The effects of the treatment with a Body Monochord and listening to relaxation music (randomized chronological presentation) on SCL and salivary cortisol in relation to the emotional valence of the experience were examined in patients with psychosomatic disorders (N = 42). Salivary cortisol samples were collected immediately before and after the expositions. Subjective experience was measured via self-rating scales.

Overall, both the exposure to the Body Monochord as well as the exposure to the relaxation music induced an improvement of patients’ mood and caused a highly significant reduction of SCL. A more emotionally positive experience of relaxation correlated with a slightly stronger reduction of the SCL. Both treatment conditions caused a slight increase in salivary cortisol, which was significant after exposure to the first treatment. The increase of salivary cortisol during a relaxation state is contrary to previous findings. It is possible that the relaxation state was experienced as an emotional challenge, due to inner images and uncommon sensations that might have occurred.

## Introduction

Listening to music can promote a subjective feeling of wellbeing and induce a psychophysiological relaxation response, which is characterised by a reduction of the sympathetic activity of the autonomic nervous system. Various musical interventions in clinical settings were able to induce reductions of heart rate, blood pressure and respiratory rate, as well as decreases in anxiety and improvements of mood in different patient groups [1–8]. Especially slow and soothing music seems to facilitate the vegetative relaxation effect [9–12].

The electrodermal activity (EDA) denotes changes in the electrical skin conductance and is regarded as an indicator for changes in the arousal of the autonomic sympathetic nervous system. The tonic Skin Conductance Level (SCL), which comprises slow spontaneous fluctuations of the sympathetic innerved eccrine sweat gland activity, is regarded to be a valid parameter for a relaxation reaction and is considered to be an indicator for general arousal [13]. Moreover, the EDA is considered to be an indicator for emotion-associated sympathetic activity. Various studies have shown a relation between the reduction of tonic SCL and the induction of pleasant emotions, like contentment (described as feelings of wellbeing, relaxation and peacefulness) and feelings of safety. Unpleasant emotions like anger, anxiety, fear and disgust are associated with an increase in tonic SCL [14].

Aside from the activation of the sympathetic nervous system the release of cortisol, which is modulated by the hypothalamic pituitary adrenal axis, is a further indicator of the psychophysiological stress reaction. Salivary cortisol is considered to be a valid indicator of plasma cortisol [15] and the most common biological parameter in stress research [16]. Several studies have shown a direct effect of different relaxation techniques (or methods) on the reduction of cortisol levels [17–21]. Similarly, listening to music has modulating effects on the release of cortisol. For instance, meditation music was associated with a reduction of cortisol levels [22], listening to music after inducing a stress reaction led to a stronger decrease of cortisol levels and listening to music during invasive medical interventions was associated with a lower increase of cortisol levels [23, 24].

As described in detail previously [25], the treatment with a monochord, especially the Body Monochord is used as body-oriented music therapy, which uses vibroacoustic stimulation, which can induce the experience of deep relaxation states and feelings of altered body perception [26–28]. The Body Monochord consists of a wooden resonance box standing on four feet, on which the patient lies during the treatment session. Below the resonance box there are various strings (tuned in fifth tuning). The strings are played by the therapist sitting beside the Body Monochord. By stroking evenly across the strings with fingers of both hands a sound carpet with a distinct emergence of overtones is created, whereas familiar musical structural parameters like rhythm, melody or changes in harmony are missing. In addition, there’s a sensory stimulation of the patient’s body by the vibrations due to the direct physical contact with the resonance box. Body Monochords can have different shapes, forms, and harmonical tunings (e.g. fifths, octave or identical tuning) [25]. Research on therapeutic application of monochord sounds and vibroacoustic stimulation via Body Monochord has been conducted with oncological patients [29, 30] and palliative care patients [31, 32]. Positive effects like reduction of anxiety and improvement of wellbeing [29, 30] as well as relaxing and calming experiences were reported [31].

In this study we examined whether vibroacoustic stimulation through a Body Monochord differs from listening to relaxation music from audio CD concerning the psychophysiological relaxation response as it is shown by tonic electrodermal activity and salivary cortisol levels in patients with psychosomatic disorders. As only little research has been conducted on the treatment with a Body Monochord in clinical subjects, this study is part of a larger study, whose other research topics deal with qualitative and quantitative interview data on subjective experience and electrocortical activity of the brain during the exposure to a Body Monochord, derived from various groups of psychosomatic patients [25, 33].

Focusing one’s attention and abstaining from goal-oriented analytical thinking has been discussed as necessary for experiencing a state of relaxation [34, 35]. Additional stimulation by vibrations in treatment with the Body Monochord possibly induces a stronger focussing of attention and thus a deeper relaxation state than merely listening to familiar relaxation music. It was shown that music combined with tactile perception of low frequencies had a stronger effect on the reduction of blood pressure and on the improvement of wellbeing in depressive patients than the mere auditory perception of music [36]. The frequency spectrum of the Body Monochord used in this study also covers the low frequency region, as there are two sets of three strings which are tuned to the tone pitches of A1 (55 Hz) and D1 (36.7 Hz). Thus, we expected a stronger reduction of tonic EDA and salivary cortisol levels during the exposure to the Body Monochord than by listening to CD music.

## Methods and Material

### Subjects

The sample consisted of 42 patients (26 of which were women) aged between 20 and 76 (M = 48.5, SD = 12.2) with psychosomatic disorders (somatoform disorder: N = 15; adjustment disorder: N = 11; depressive disorder: N = 11; anxiety disorder: N = 5). The patients participated in the study during inpatient treatment at the Department for General Internal and Psychosomatic Medicine at Charité—Universitätsmedizin Berlin. The study was approved by the ethics committee of Charité–Universitätsmedizin in Berlin (application number: EA1-290-12) and informed consent was obtained from all patients. All patients provided their written informed consent to participate in this study.

### Procedure

Each patient received a 20-minute exposure to a Body Monochord and a 20-minute presentation of slow consonant relaxation music (panpipe with piano accompaniment), which was played on an audio CD. The strings of the Body Monochord were tuned in fifths tuning (tone pitches: D3, A2, D2, with additional A1 and D1 at three strings each), which allowed it to emit a sound with a manifold overtone spectrum. The Body Monochord was constructed by the manufacturer of musical instruments Bernhard Deutz in Berlin (http://www.deutz-klangwerkstatt.de; a sound sample is available at http://psychosomatik.charite.de/forschung/koerpererleben_koerperzentrierte_therapieverfahren/). For clinical reasons (practicability) data acquisition took place from about 11 a.m. to 12 p.m. Immediately before each exposure the patients were lying in a quiet resting state for two minutes. Both treatments were presented in succession in randomized order, with a break of about ten minutes in between. While listening to the CD music patients were also lying on the Body Monochord. The patients received the instruction to close their eyes and do nothing else aside from listening to the sound of the music. Immediately after each treatment method two self-rating scales for assessing the subjective experience during the single treatment exposition and the state of mood after the treatment were presented.

### Cortisol Measurement

Saliva cortisol samples were collected directly before the first treatment session (T1), immediately after the first one (T2) and immediately after the second treatment session (T3), using Salivette® sampling devices (Sarstedt, Germany). On doing so the patients chewed on a cotton roll for the duration of about two minutes. The samples were immediately chilled on ice and centrifuged at 2400 x g for 2 minutes at 4°C. Then the samples were chilled on ice again and after the procedure of the presentation of the two treatment methods they were frozen in aliquots at -80°C until assayed. Cortisol levels (nmol/l) were determined using the commercially available immunoassay ELISA-Kit Parameter™ Cortisol Assay (R&D Systems, Inc., USA). According to the manufacturer the assay sensitivity for the kit was 0.111 nmol/l. Intra-assay and inter-assay coefficients of variation were 5.4% and 9.3%, respectively.

### EDA Measurement

During the whole investigation SCL was recorded continuously exosomatically from the surface of the skin via application of a direct current voltage of 1.5 V, using the portable biofeedback device MentalBioScreen K3 (Porta Bio Screen GmbH, Germany). For the measurement EKG foam electrodes with a carbon push button and adhesive gel pad (43 x 45 mm) were used (ASF-40C, Bio Protech Asmuth GmbH, Germany). Two electrodes were fixed to the hypothenar site on each palm of both hands, overlapping about 0.5 cm, thus the distance was about 3.5 cm. The subsequent EDA-analysis was conducted for a 1-minute interval during the resting position before the treatments and for 1 minute intervals during the periods of 0–1, 5–6, 11–12 and 17–18 minutes following the start of the Body Monochord and CD music exposure. The SCL values were averaged for each time interval.

### Subjective Experience

As described in detail previously [25], the subjective experiences that occurred during the exposure to the Body Monochord and the exposure to the CD music were immediately recorded after the presentation of each treatment method by means of a self rating scale (7-point Likert scale). The questions were based on the dimensions of experience of the Phenomenology of Consciousness Inventory (PCI) by Pekala [37], German version by Rux [38], a questionnaire for assessing altered states of consciousness, which can arise e.g. during relaxation states [39]. For assessing the positive emotional feelings during the two treatments, patients were asked about feelings of joy, feelings of kindness and the feeling of safety that occurred during the treatment methods. For each subject the mean value of these categories was calculated separately for each treatment method. Since negative feelings like fear, anger, sadness showed rather low values and differed only very slightly between patients, categories of emotionally negative response were not included in this assessment [25].

Additionally, the Berlin Mood Questionnaire (Berlin Mood Scale, BMS) [40] was carried out at three points of cortisol measurement (T1, T2, T3). The 30-item BMS measures six different states of mood (listlessness, tiredness, anxious depression, anger, involvement, elevated mood). The items are presented on a 5-point Likert scale.

### Statistical Analysis

Statistical data analysis was performed using the statistical software SPSS (Version 20). We tested the possible effects of the chronological order of the single treatment methods and the kind of treatment on positive emotional feelings. We did so by calculating an ANOVA with repeated measurement of the within-subject factor ‘kind of treatment’ (Body Monochord, CD music) and the between-subject factor ‘treatment chronological order’ (Body Monochord first, CD music first). Differences in the various dimensions of the BMS in depending of the different treatment conditions (T1, T2, and T3) were tested by calculating t-tests for dependent samples.

The possible influences of the chronological order and the kind of the treatments on the SCL were tested by calculating an ANOVA with repeated measurement of the factor ‘kind of treatment’ (Body Monochord, CD music) and ‘point in time of measurement’ (Rest, 0–1 min, 5-6- min, 11–12 min, 17–18 min) and the between-subject factor ‘treatment chronological order`(Body Monochord first, CD music first).

For statistical analysis of the Cortisol data an ANOVA with repeated measurement of the factor ‘time of measurement’ (before 1^st^ treatment, after 1^st^ treatment, after 2^nd^ treatment) and the between-subject factor ‘treatment chronological order’ (Body Monochord first, CD music first) was calculated. The specifications for alpha error of variance analytical significance test were based on the Greenhouse-Geisser-adjusted degrees of freedom. Multiple comparisons in t-tests were adjusted using the Bonferroni-method or Bonferroni-Holm-method, when more than three comparisons were conducted. Effect sizes were indicated with the coefficients Partial Eta Squared (η^2^) and Cohen’s d.

## Results

Six patients were excluded from the statistical analysis of the salivary cortisol data, because their measurement values of salivary cortisol were outside the standard curve. One patient was excluded from the statistical analysis of the self-rating scale and five patients were excluded from the statistical analysis of the BMS because of missing measurement data. SCL data of eight patients could not be used for statistical analysis because of measurement errors due to heavy perspiration and low adherence of the electrodes.

### Subjective Experience

**Fig 1:**
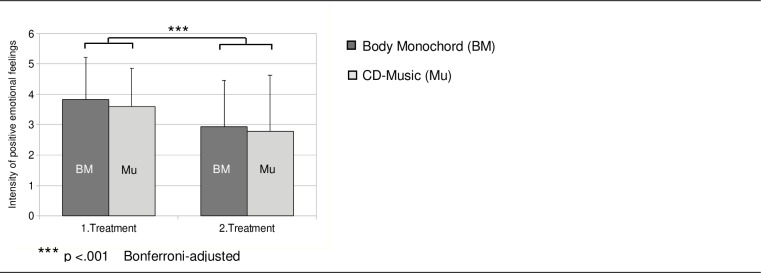
Positive emotional feelings. Mean values of the positive emotional feelings (feelings of joy, feelings of kindness and the feeling of safety) during the treatments with the Body Monochord (BM) and CD-music in context of the chronological order of the presentation.

Results revealed a significant interaction ‘kind of treatment x treatment chronological order’ (F[1,39] = 4.33; p = .044; η^2^ = .100). Thus, the treatment method, which was chronologically presented first, was emotionally experienced in a significantly more positive way than the second treatment (Fig 1). The factor ‘kind of treatment’ did not differ significantly in the positive emotional feelings during Body Monochord and CD music exposure.

**Fig 2:**
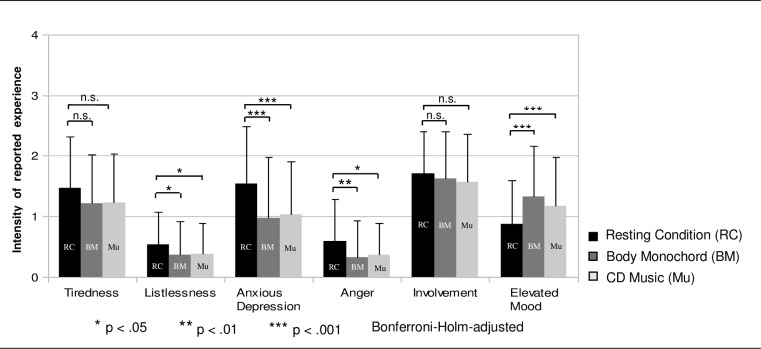
Berlin Mood Questionnaire (BSF). Perceived states of mood measured via the Berlin Mood Questionnaire (BSF) at the resting condition, after the exposure to the Body Monochord, and after the exposure to the CD music. (N = 37).

The statistical analysis of the BMS showed a significant increase of elevated mood (Body Monochord: t = -4.53; df = 36; p = .00062; Cohen’s d = -.76; CD music: t = -4.64; p = .00068; Cohen’s d = -.73; Bonferroni-Holm adjusted) and significant reductions of listlessness (Body Monochord: t = 2.95; df = 36; p = .034, cohen’s d = .48; CD music: t = 2.97; p = .037; df = 36; Cohen’s d = .49), anger (Body Monochord: t = 3.53; df = 36; p = .009; cohen’s d = .59; CD music: t = 2.78; df = 36; p = .045; Cohen’s d = .46), and anxious depression (Body Monochord: t = 5.75; df = 36; p = .000025; Cohen’s d = .96; CD music: t = 4.85; df = 36; p = .00026; Cohen’s d = .82), both after the exposure to the Body Monochord and after the exposure to the CD music. All significance values were Bonferroni-holm adjusted. No significant differences were found between the two kinds of treatment (Fig 2).

### Salivary Cortisol

**Fig 3:**
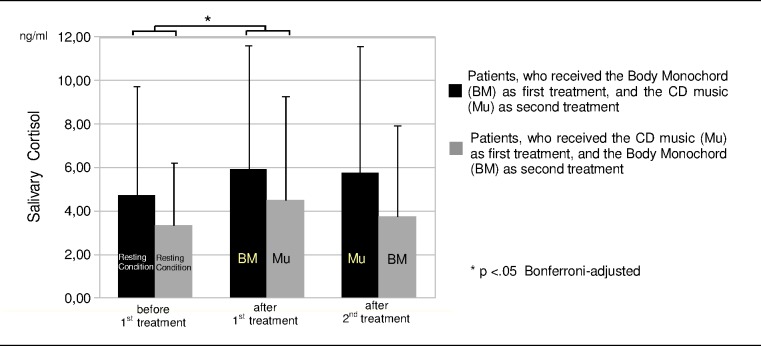
Salivary cortisol. Salivary cortisol levels measured immediately before the first treatment after the resting condition, immediately after the first treatment and after the second treatment for the two groups with different chronological order of the two kinds of treatment (N = 36).

After the presentation of the chronologically first treatment an increase in salivary cortisol level could be observed both for the Body Monochord and the CD music condition, which slightly decreased after the second treatment presentation (Fig 3). The factor ‘time of measurement’ (before 1st treatment, after 1st treatment, after 2nd treatment) was significant (F[2,68] = 3.332; p = .051; η^2^ = .089). The direct comparison of the mean values between the three points of measurement revealed that the increase of the salivary cortisol level from the first to the second point of measurement is significant (t = -2.688; df = 35; p = .033, Cohen’s d = .24; Bonferroni-adjusted). The difference of salivary cortisol between the first and the third point of measurement missed the significance level (t = -1.404; df = 35; p = .169, Cohen’s d = .15), as well as the difference between the second and the third point of measurement (t = 1.156; df = 35; p = .256, Cohen’s d = .07).

The between subject factor ‘treatment chronological order’ did not show any significance, which means that the cortisol levels did not differ significantly between the patients, who were presented the two treatment conditions in chronological different order.

The interaction ‘time of measurement x treatment chronological order’ was also not significant, revealing that chronological course of the cortisol levels did not differ significantly in dependence of chronological order of the treatment presentation. Thus the two treatment methods Body Monochord and the exposure to CD music did not differ significantly in their influence on the secretion of cortisol. As well, the direct comparison of the mean values between Body Monochord and CD music did not show any significance.

As the increase in cortisol levels was contradictory to our expectations, we explored possible artefacts. For all patients the last medication and food intake took place between 8 a.m. and 8.30 a.m., about 3 hours before the psychophysiological measurements. Six patients did not take any medication at all, whereas thirty patients took one or more medicaments of the medication listed in Table 1. As cortisol belongs to the class of glucocorticoids, the intake of glucocorticoids might have influenced the cortisol system. The other drug agents listed in the table are not known to significantly influence cortisol levels. Furthermore, it has been shown that underweight and obesity have various influences on patients’ cortisol levels and responsiveness of the cortisol system [41, 42]. For that reaseon we repeated the calculation of the compared t-test without those patients, who had taken glucocorticoids (N = 5) and those patients with a BMI over 30 (N = 5) or below 18 (N = 3), so that altogether 13 patients were excluded. The results of the new calculation confirmed the previous findings and revealed even a higher effect size for the increase in salivary cortisol levels from the first to the second point of measurement (t = -2.58; df = 22; p = .051, Cohens d = .36, Bonferroni-adjusted). The differences in salivary cortisol between the first and the third and between the second and the third point of measurement again missed the significance level.

**Table 1:** Medication, taken by the patients about 3 hours before physiological measurements.

Drug class	Agent
Antidepressant	Citalopram, Sertralin, Duloxetin, Amitriptylin, Trimipramin, Opipramol, Bupropion, Mirtazapin, Hypericum perforatum
Antiepileptic	Gabapentin
Neuroleptic	Olzapin, Pipamperon
Sedative	Zoplicon
Antidiabetic	Metformin
Cholesterol reducer	Simvastatin, Ezetimib
Antihistamine	Mizolastin, Fexofenadin, Dimetinden, Betahistin
Proton pump inhibitor	Omeprazol, Pantoprazol, Lansoprazol
Alpha-blocker	Doxazosin, Tamsulosin
ACE inhibitor	Enalapril, Ramipril, Lisinopril
Beta-blocker	Metoprolol, Bisoprolol
AT1-antagonist	Candesartan, Olmesartan, Telmisartan
Calcium channel blocker	Amlodipin, Lercanidipin
Non-steroidal antirheumatic/non-opioid analgetic	Diclofenac, Acetylsalicylic acid, Metamizol, Flupirtin, Paracetamol, Ibuprofen
Glucocorticoid	Fluticason, Triamcinolon, Methylprednisolon, Budesonid, Ciclesonid
Vitamin	Thiamin (Vitamin B1), Colecalciferol (Vitamin D)
Mineral	Magnesium, Kaliumiodid, Dinatriumhydrogenphosphat, Kalium dihydrogenphosphat, Calcium carbonat,
Diuretic	Hydrochlorothiazid, Torasemid
Immunosuppressant	Everolimus, Mycophenolat
Sex hormon	Estradiol
Thyroid hormone	Levothyroxin
Spasmolytic	Darinefacin
Dopamine agonist	Pramipexol
Antiemetic	Metoclopramid
Thrombocyte aggregation inhibitor	Clopidogrel
Beta-sympathomimetic	Salbutamol

### EDA-Activity (SCL)

**Fig 4:**
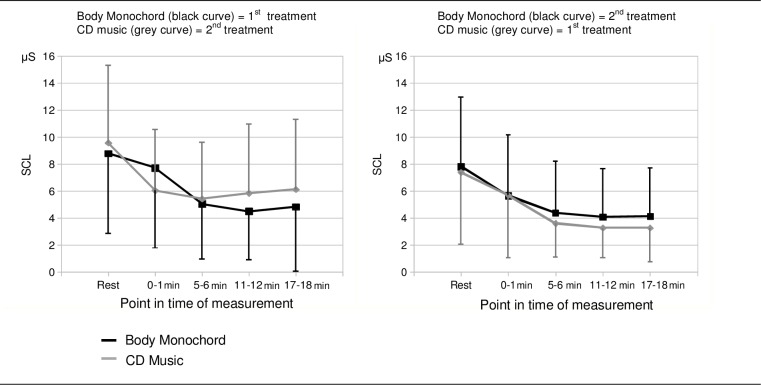
Electrodermal activity (SCL). Course of SCL during the two kinds of treatment (Body Monochord, CD music). The left diagram shows the courses of the SCL when the Body Monochord was presented as first and the CD music was presented as second exposure. The right diagram shows the courses of the SCL when the CD music was presented as first and the Body Monochord was presented as second exposure.

During both treatment methods a pronounced decrease of SCL was observed (Fig 4). The factor ‘point in time of measurement’ was highly significant (F[4,128] = 31.59; p < .001; η^2^ = .497) and showed a highly significant linear trend (F[1,32] = 42.38; p < .001; η^2^ = .570. The factor ‘kind of treatment’ was not significant. These results indicate that the decrease of SCL during the two treatment methods was significant over time and did not differ significantly between the two treatment methods. We found a highly significant interaction effect between ‘kind of treatment’ and ‘treatment chronological order’ (F[1,32] = 15.15; p < .001; η^2^ = .321). This indicates that during the treatment method, which was presented first, there was a greater reduction of SCL compared to the treatment method, which was presented in the second place.

The interaction ‘kind of treatment x treatment chronological order x point in time of measurements” reached only the 10% alpha error level (F[4,128] = 2.98; p = .067; η^2^ = .085), but shows the tendency for difference between the two treatment methods in the course of SCL over time dependent of the chronological order of presentation. Thus, we calculated the mean values of the last three measurement points (5–6, 11–12 and 17–18 min, following the start of the treatments) separately for each treatment presentation and performed paired t-tests to check the significance of the differences between the treatment method that was presented first and the treatment method that was presented second. Results revealed that during the treatments presented first, there were higher decreases in SCL than during the treatments presented second, which only just missed the significance level of 5% (Body Monochord presented first: t = 2.34; df = 20, p = .060, cohen’s d = .45, Bonferroni-adjusted; CD music presented first: t = 2.43; df = 16; p = .054, cohen’s d = .63, Bonferroni-adjusted).

## Discussion

In this study we examined the effects of vibroacoustic stimulation via a Body Monochord in comparison with a relaxation music CD on the skin conductance level (SCL) and salivary cortisol levels in patients with psychosomatic disorders, in relation to the emotional evaluating scores for subjective experience during the exposure to the treatments.

### Subjective Experience

As a sequence effect we found that the treatment mode presented first was scored in an emotionally more positive way (respectively, feeling of kindness, feeling of safety, and feeling of joy) than the treatment mode presented in the second place, a result which corresponds to the findings of a larger sample without cortisol measurements [25]. This may be explained by the duration of the examination, taking about two hours altogether, which might have reduced patients’ willingness to get involved in the second treatment. Furthermore, both the exposure to the Body Monochord and the exposure to the CD music induced an improvement of patients’ scores for state of mood, namely an increase in elevated mood and a decrease in anxious depressiveness, anger and listlessness.

### Salivary Cortisol

Both after the presentation of the first and the second treatment session, an increase in salivary cortisol was observed both for the exposure to the Body Monochord and the exposure to the CD music. The results revealed that the increases from the initial resting period to the points of measurement immediately after the first treatment exposure were significant. The treatment with the Body Monochord and the treatment with the CD music did not differ significantly in their influence on the secretion of cortisol. In visual terms, the group of patients, who had been presented the CD music at first, showed lower levels of cortisol at all the three measurement points, but these differences did not show any significance. The lack of significance can be explained by the high standard deviations of the measurement values. For the same reason the increase between the first and second measurement points was significant and the increase between the first and the third measurement points was not.

The increase of salivary cortisol levels during the two treatments with the Body Monochord and the CD music is contrary to our expectations that listening to music, especially to relaxation music, and experiencing relaxation states are associated with a decrease in cortisol levels, as revealed in former studies [17–19, 21– 23, 43, 44]. After the first treatment session, which was experienced in an emotionally more positive way, the increase in salivary cortisol was significant and more pronounced than after the second presentation of the treatment session, which was emotionally experienced in a slightly less positive way.

Regarding research on the relation between emotion, affect and cortisol, an increase in cortisol levels is in general associated with negative affect. On the other hand, positive affect is rather associated with a decrease in cortisol levels [45–47]. Other studies revealed that there was no effect of positive daily events on cortisol levels [48]. Furthermore, it was shown that positive emotion with high physiological arousal, in terms of being alert and active, was associated with a decrease in salivary cortisol [49]. On the other hand, positive emotion with low physiological arousal, in terms of being happy and relaxed, did not show any effects on cortisol levels.

Thus, our results seem to contradict findings of studies, which revealed that negative, but not positive affect is associated with higher cortisol levels. Another investigation of our working group, which compared the effects of the Body Monochord in the shape of a chair and in the shape of a lounger, found similar results concerning the increase of salivary cortisol levels in patients suffering from eating disorders, somatoform disorders and pain disorders [50]. This makes it unlikely that the reported increase in salivary cortisol is based on measurement errors.

It might be possible that the relaxation states, induced by listening to the relaxation music and by vibroacoustic stimulation, in some patients also constituted a kind of emotional challenge related to stress, which might be reflected in the increase of cortisol levels. Relaxation techniques, which use imagery, are supposed to be more likely to induce adverse experiences than relaxation techniques without imagery, because intrusive distressing images and thoughts might be enhanced more easily through imagery instructions, which would lead to an increase in arousal [51]. In general, during relaxation states unconscious or preconscious contents, which might be related to conflicts in one’s personal life, are more likely to appear [52]. The appearance of these phenomena is used in the psychotherapeutic technique e.g. of Guided Imagery and Music [53].

The analysis of qualitative interview data on subjective experiences during the treatment with a Body Monochord of patients suffering from eating disorders [54] revealed that changing inner images, thoughts, body-related feelings and emotions occurred during the course of the treatment, which also means that short phases of pleasant and unpleasant experiences sometimes alternated. Possibly, similar subjective experiences during the treatment with the Body Monochord and CD music might have occurred in the patients we investigated in this study.

Previous studies on the impact of music or relaxation on cortisol levels mainly investigated healthy subjects who were not suffering from psychological disorders [22, 23, 43, 55]. As the patients in our study were suffering from depressive disorders, anxiety disorders, adjustment disorders and somatoform disorders, which are supposed to be associated with individual psychosocial stress factors, part of these stress factors might have come to consciousness by focusing the attention towards the self by means of the vibroacoustic stimulation and listening to the sound and music. Taken as a whole, aside from pleasant and relaxing phases of subjective experiences during the treatment sessions, the treatments by themselves might have been emotionally challenging experiences, which could be the reason for the increase of patients’ salivary cortisol levels.

The former results of the analysis of the electrocortical activity (EEG) during the treatment with the Body Monochord in a larger sample [33] might give a hint to a possible mental stress processing in the beginning of the treatment. During the first minutes of exposure to the Body Monochord a desynchronsation both of the EEG-Theta and Alpha bands occurred (see results in [33]). As described in detail previously [33], desynchronised EEG-Alpha activity is an indicator of increased attention during the expectation of cognitive tasks and information processing [56, 57]. Synchronised Theta activity is associated with relaxation [58] (and correlates with focused attention and memory search [59–61]. Due to the fact that both the Alpha and the Theta bands are desynchronisized, this result suggests that in the beginning of the exposure to the Body Monochord a state of increased vigilance took place without any mental relaxation, memory processing or focused attention. The desynchronization of the EEG could thus be matched with a situation of mental orientation in which an attempt is made to make sense of and to structure the unusual perception, which might also be interpreted as stress processing. It should be mentioned that during the exposure to the CD music these EEG effects were not observed.

### Skin Conductance Level

The increase in salivary cortisol as an indicator of a stress response seems to be contradicted by the decrease in the SCL, which indicates an experience of relaxation. During the first treatment the decrease in SCL was significantly more pronounced than during the second treatment. This result corresponds with the sequence effect of positive emotional feelings, which were scored as being higher during the first treatment compared to the second treatment. This is consistent with results of former studies, which demonstrated an association between feelings of relaxation, wellbeing or contentment and a reduction of SCL [14].

As we discuss the increase in salivary cortisol levels as a possible indicator of a stress response that occurred during the treatment sessions, and as the SCL is regarded to be an indicator of emotion-associated sympathetic activity, one would expect an increase in SCL during an emotionally challenging experience. But the emotionally associated SCL response is probably masked over by a possible relaxation response, which is induced by focussing on the music or by just lying on the lounger. In a study by Nater and colleagues [62] on psychophysiological and emotional responses to musical stimuli, listening to Heavy Metal music led to an increase in unrest and aggression, whereas smoothing classical music led to more calmness and improvement of mood. The SCL levels were higher during listening to Heyvy Metal music, but both during the Heavy Metal music and the relaxing music a decrease in the courses of SCL could be observed. However, these contradictory results were not discussed in this article, because the focus of the study was on different responses of men and women to musical stimuli. Possibly the focussing on the music in a resting position facilitated a physical relaxation state, which at the same time was masked by the emotional arousal because of the music. Thus, on the one hand during listening to Heavy Metal music the SCL was higher than during relaxation music, but on the other hand the SCL decreased during both conditions probably due to physical relaxation.

**Fig 5:**
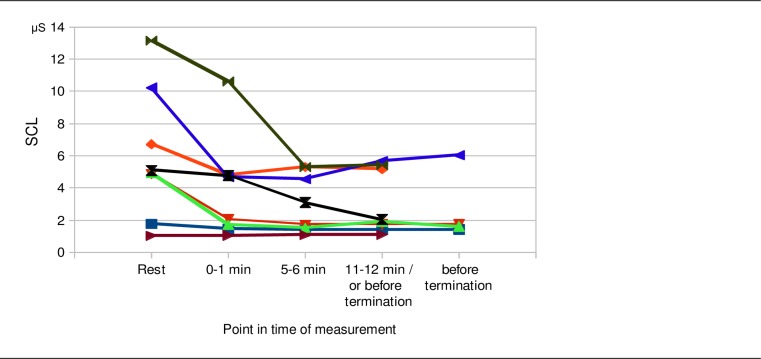
Electrodermal activity (SCL) of the patients who discontinued the treatment session. Individual courses of the SCL of the patients who discontinued the treatment session with the Body Monochord (N = 7) or the CD music (N = 1) because of unpleasant feelings or occurring imageries. The last measurement points refer to the last minute immediately before termination of the treatment exposure.

It should also be noted that in our study on the effects of the Body Monochord and relaxation music on psychophysical parameters and subjective experience we altogether examined 101 patients (of which only 42 were measurd by salivary cortisol) of which 8 patients discontinued the treatments because of unpleasant feelings or imageries that occurred (Body Monochord: N = 7, CD music: N = 1). Fig 5 shows the courses of SCL until the termination of the treatment. It is striking that 6 patients showed an obvious decrease in SCL, whereas in 2 patients the SCL remained about the same level. These results give hint to the existence of a kind of paradoxical relaxation response, in which the electrodermal activity decreases, whereas unpleasant or stressfull feelings or imageries are processed mentally. The physical position of resting and lying down probably facilitates the relaxation of the skeletal muscles. The patients were invited to do nothing else but paying attention to the sound and music. Focusing of attention without distraction combined with a relaxing position of the body is supposed to be necessary to encourage the appearance of a relaxation response, as described in the literature [34, 35].

However, the question arises why the increase in cortisol was not masked by the same effect. Possibly the electrodermal activity and the activation of the HPA-axis represent independent components concerning the relaxation response. This could mean that the decrease in SCL might be more strongly influenced by the physical calming effect whereas the increase in salivary cortisol levels might be more associated with inner mental activity and might be less sensitive to mere physical rest. A study of Pawlow and Jones [63] showed that merely sitting quietly did not show any impact on salivary cortisol levels.

However, the exposure both to the Body Monochord and to the relaxation music led to an increase in elevated mood and a decrease in anxious depressiveness, anger and listlessness (dimensions of the BMS–Berlin Mood Scale). It should be noted that the self-rating of the BMS refers to the state of mood that patients experienced at the point in time immediately after the treatment exposure. Probably the state of mood did not remain the same during the whole exposure. Presumably, during the exposure to the Body Monochord and maybe also during the CD music changing contents of mental images, thoughts and memories ocurrued and both pleasant and unpleasant states of mood might have appeared alternately, similar to the patients suffering from eating disorders, whose subjective experience during Body Monochord treatements was investigated in another study of our working group [54]. The viewing of the qualitative interview data of patients in the present study and of the whole sample suggests that part of the patients indeed experienced the treatment as emotionally challenging, especially in the beginning. This happened because unpleasant memories or actual problems in life emerged in their minds, from which, however, they could distance themselves in the further course of the treatment exposure [64]. Thus, the rated mood at the point in time after the treatment exposure might be the result of coming to terms with a somehow challenging emotional process.

Another explanation for the increase in salivary cortisol might be the possibility that the patients’ experiences of elevated mood and decrease in listlessness also indicated an activating process, induced both by the bodily vibroacoustic stimulation and the inner images, which might have been encouraged through sound and music. So this activating process might have been experienced as a kind of eustress, which might have caused the increase of salivary cortisol levels.

### Limitations

The cortisol results must be interpreted with caution, because it cannot be completely excluded that the release of cortisol was influenced by a variety of drugs like beta-blocker propanol, antidepressants or others, which were taken by various patients prior to testing. Possibly the medication caused a delayed onset of the cortisol release and led to the rise in cortisol observed over the treatment.

Another limitation regarding cortisol might be the fact, that the release of cortisol follows a circadian rhythm, which culminates in the morning after waking up and decreases throughout the rest of the day [65, 66]. The patients had been tested during the late morning when higher fluctuations in cortisol levels can be assumed. To address this problem and to reduce possible influences of medication, further studies should be conducted in the late afternoon.

As the focus of this study was on the impact of vibroacoustic stimulation on patients’ salivary cortisol levels and electrodermal activity in relation to their subjective emotional experience, we did not include a control group with healthy subjects. Thus the study has an explorative character and the absence of a control group can be considered as a limitation.

Furthermore, it can be regarded as a limitation that we did not include more direct psychological measures of relaxation and stress e.g. like visual analogue scales, because the focus of the study was on the patient’s state of mood and emotional experience. This could have provided additional information about the perceived state induced by the treatments.

A further limitation of the study is the fact that there was only a break of ten minutes between the successive interventions. This might have been not enough time for the physiological systems to return to a true baseline. One way of avoiding this problem is to perform the measurements on two different days. But it must be taken into consideration that on different days usually different unavoidable therapeutic and diagnostic procedures are conducted due to the clinical context, which might have been experienced as different additional stress factors. In this context our study has an explorative character, which should invite to further studies.

Finally, it can be taken into consideration whether the cortisol system of patients might be dysregulated to varying degrees due to long lasting distress, which finally led to disease and inpatient admission. Moreover, it is to be assumed that patients had different lengths of time for recovery, because of different length of hospital stay or different influencing factors which might have occurred during the stay in hospital before the investigation. This might have some impact on possible differences between the responsiveness of the cortisol system and the electrodermal activity to pleasing or displeasing stimuli. In order to address this issue, it would be necessary to investigate several treatments in single persons over a longer period of time by means of time series analysis.

## Conclusion

A relaxation state, induced by vibroacoustic stimulation or listening to CD music, is associated with a reduction of the electrodermal activity and thus of the sympathetic arousal, which was more pronounced during higher emotionally positive feelings during the treatments.

Both vibroacoustic stimulation and listening to relaxation music induced an improvement of patients’ mood and were associated with an increase in salivary cortisol. The increase in salivary cortisol might be explained by a potentially challenging experience of the relaxation state due to possibly uncommon body sensations and inner images. Thus the electrodermal activity and the HPA-axis might be independent components concerning the relaxation response, which could mean that the decrease in tonic electrodermal activity might better reflect the physical calming effect and the increase in salivary cortisol levels might be more associated with inner mental activity.

## Supporting Information

S1 File Patients’ data. The data file contains patients’ data of Sex, Age, Salivary Cortisol, Electrodermal Activity (SCL), Positive Emotional Feelings and Berlin Mood Scale (BMS).
(XLS)

S2 File SCL of dropouts. Electrodermal Activity (SCL) of patients, who discontinued the treatment exposure.
(XLS)
